# The DC–T cell axis is an effective target for the treatment of non‐small cell lung cancer

**DOI:** 10.1002/iid3.1099

**Published:** 2023-11-27

**Authors:** Shuangcui Wang, Guan Zhang, Qian Cui, Yanjie Yang, Dong Wang, Aqing Liu, Ying Xia, Wentao Li, Yunhe Liu, Jianchun Yu

**Affiliations:** ^1^ Department of Oncology First Teaching Hospital of Tianjin University of Traditional Chinese Medicine Tianjin China; ^2^ National Clinical Research Center for Chinese Medicine Acupuncture and Moxibustion Tianjin China; ^3^ Graduate School Tianjin University of Traditional Chinese Medicine Tianjin China

**Keywords:** artificial DCs, chimeric antigen receptor T cell immunotherapy, DCs, NSCLC, T cells

## Abstract

The dendritic cell (DC)‐T cell axis is a bridge that connects innate and adaptive immunities. The initial immune response against tumors is mainly induced by mature antigen‐presenting DCs. Enhancing the crosstalk between DCs and T cells may be an effective approach to improve the immune response to non‐small cell lung cancer (NSCLC). In this article, a review was made of the interaction between DCs and T cells in the treatment of NSCLC and how this interaction affects the treatment outcome.

## Graphical abstract

DC–T cell axis is an effective target for treating non‐small cell lung cancer.

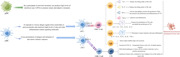

## 1 INTRODUCTION

Lung cancer is a prominent cause of death related to cancer across the globe.^1^, ^2^, ^3^, ^4^ The projected rise in its incidence and mortality rates in the upcoming years presents an ongoing public health risk.^5^ From a histopathological perspective, lung cancer has two major classifications: small and non‐small cell lung cancers (SCLC and NSCLC, respectively). The latter accounts for around 85% of all lung cancer cases, with a 5‐year overall survival (OS) rate of about 15%.^6^, ^7^, ^8^ Thus, it is of particular importance to identify more critical factors influencing the treatment efficacy of NSCLC.

For the past few years, applying tumor immunotherapy has turned into a key topic of research.^9^ At present, immune checkpoint inhibitors (ICIs) are considered a special class of tumor immunotherapy drugs. At the late stage of tumors, metastasis is usually accompanied by brain, bone, liver metastases, and so forth. ICIs are also important in treatment. Tumor mutation burden and programmed death receptor ligand‐1 and 1 (PD‐L1 and PD‐1, respectively), as well as the number and percentage of lymphocytes, are biomarkers for the occurrence of metastasis in NSCLC. PD‐1 and PD‐L1 inhibitors are most studied and applied currently, and have good therapeutic effects in patients with brain,^10^, ^11^, ^12^ liver,^13^, ^14^ and bone metastases.^15^ Primarily, ICIs assist immune cells in eliminating tumor cells by obstructing their ability to evade the immune system, which thus amplifies their antitumor impact.^16^ In tumor cells and tissues, PD‐L1 binds to the tumor‐infiltrating lymphocyte PD‐1. This interaction suppresses the reactivity of effector T cells, which enables tumor cells to evade immune surveillance by lymphocytes.^17^, ^18^ In addition, the blockade of PD‐1 enhances the natural killer (NK) cell‐mediated lysis of NSCLC, which increases the killing effect of NK cells.^19^ Moreover, cytotoxic T‐lymphocyte‐associated protein‐4 (CTLA‐4) competitively hampers the binding of the cluster of differentiation (CD28) molecules on the surface of T cells to antigen‐presenting cells (APCs). By binding to CD80 and CD86 on the surface of APCs, CTLA‐4 further impedes the function of APCs, which thus obstructs the immune process.^20^ Given that ICIs can prevent signal transduction between tumor and immune cells, they can restore the immune response against tumor cells.

Advancements in the understanding of tumor immunity have driven rapid progress in cell‐based immunotherapy techniques that target APCs and T cells. Of particular interest within this realm is the dendritic cell (DC)–T cell axis, which has been the primary focus in the pursuit of anticancer therapies. DCs are capable of initiating adaptive immune responses in T cells and directly eliminating tumor cells.^21^, ^22^ This axis connects innate and adaptive immunities and plays a crucial part in specific immunity development. It is essential to study the DC–T cell axis. Additionally, this work aimed to explore novel therapeutic opportunities that can overcome immune tolerance in NSCLC and enhance the prognosis of NSCLC patients.

## 2 CROSSTALK BETWEEN DCS AND T CELLS

### 2.1 T cells are the key to the occurrence and development of tumor immune responses

**Figure 1:**
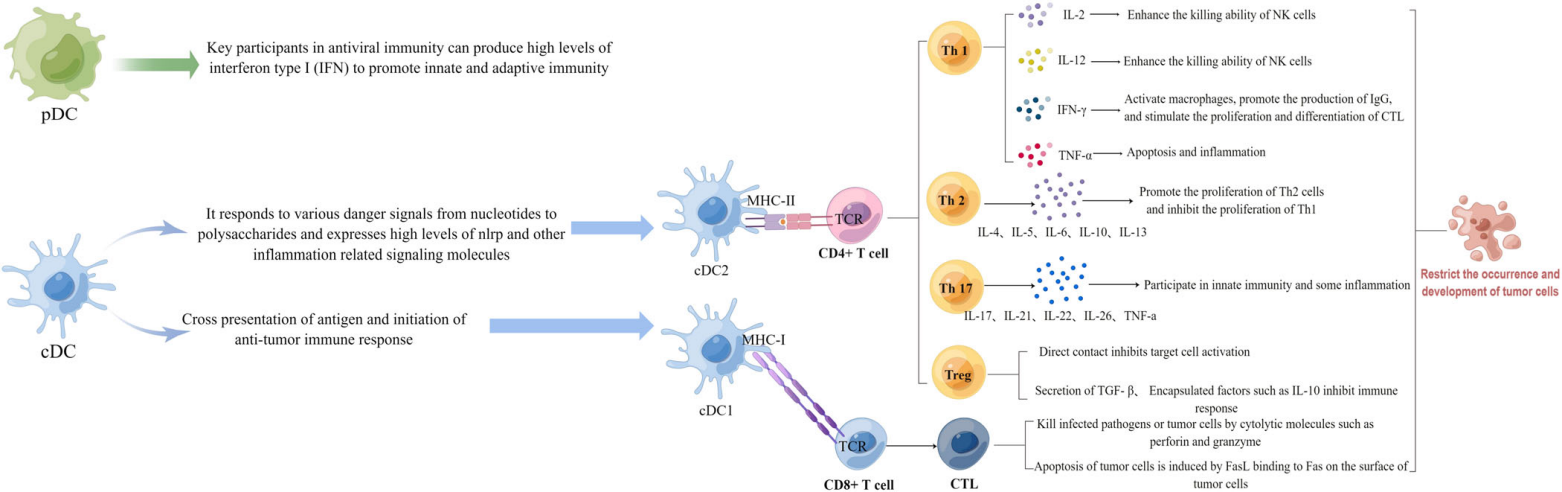
Schema chart of a complex interaction between dendritic cells and T and tumor cells.

In the thymus, T cells undergo differentiation and maturation before being disseminated to immune organs and tissues throughout the body via lymphatics. They are responsible for mediating cellular immunity and coordinating the overall immune response.^23^, ^24^ CD4^+^ and CD8^+^, two types of T cells, are particularly crucial in the development of adaptive immunity.^25^, ^26^ A major hallmark of adaptive immunity is its specificity, which is ascribed to the stimulation of specific antigens derived from tumors or pathogens. Under the influence of antigens presented by DCs, CD4^+^ naive T cells (CD4^+^ Tn) can differentiate into CD4^+^ T cells. T helper 1 (Th1), Th2, Th17, and regulatory T (Treg) cells have extensively been studied within this subset.^25^, ^27^, ^28^ Th1 primarily secretes interleukin‐2 (IL‐2), IL‐12, interferon‐γ (IFN‐γ), and tumor necrosis factor‐α (TNF‐α), which facilitates Th1 proliferation and regulates cellular immunity. In addition, these cytokines inhibit Th2 proliferation.^29^, ^30^ IFN‐γ not only activates and enhances the phagocytic properties of macrophages but also promotes the production of immunoglobulin G.^31^, ^32^ IL‐2, IL‐12, and IFN‐γ can also enhance the killing ability of NK cells.^33^ The proliferation and differentiation of cytotoxic T cells (CTLs) can be synergistically stimulated by IL‐2 and IFN‐γ. Contrarily, TNF directly triggers the apoptosis of target cells and also strengthens the inflammatory response.^34^, ^35^ Th2 primarily secretes IL‐4, IL‐5, IL‐6, IL‐10, and IL‐13 facilitating the proliferation and activation of B cells. Moreover, these cytokines induce humoral immunity while inhibiting Th1 proliferation.^36^, ^37^, ^38^ Innate immunity and inflammation are induced by Th17 through the secretion of cytokines such as IL‐17, IL‐21, IL‐22, IL‐26, and TNF‐α. Notably, Th17 promotes the development of immune‐related pathological damage, particularly autoimmune diseases.^39^, ^40^, ^41^ DCs and CD4^+^ and CD8^+^ T cells are inhibited by Treg cells during immunosuppression.^42^ This is achieved through two main mechanisms: (1) Inhibiting the capability of APCs to activate T cells, primarily through the interactions between CTLA‐4, lymphocyte activation gene‐3) on Treg surfaces and CD80, CD86, and major histocompatibility complexes (MHCs) on DC surfaces, respectively. (2) Suppressing effector T cells via the secretion of transforming growth factor‐β (TGF‐β), IL‐10, and IL‐35.^42^ Generally, the cytokines secreted by CD4^+^ T cells regulate the function of immune cells and monitor the development, differentiation, and function of CD8^+^ T, B, and NK cells, DCs, and other immune cells. CD8^+^ naive T cells (CD8^+^ Tn) can develop and differentiate into CD8^+^ T cells upon stimulation by antigens presented by DCs. Consequently, CD8^+^ T cells can differentiate into CTLs, which eliminate pathogen‐infected and tumor cells through cytolytic molecules like perforin and granzyme. Thus, this is vital for a cell‐mediated antitumor immune response.^43^, ^44^, ^45^ Alternatively, CTLs induce tumor cell apoptosis by enhancing the binding of fatty acid synthetase ligand to fatty acid synthetase on the surface of tumor cells^46^, ^47^ (Figure 1).

### 2.2 DCs are the initial factor inducing adaptive tumor immunity

DCs induce the occurrence of adaptive immunity^48^ and represent a complex heterogeneous population of APCs with significant phenotypic heterogeneity and functional plasticity. They can be split into two subpopulations: conventional and plasmacytoid DC (cDC and pDC, respectively).^49^ The further subcategories of cDCs are types 1 and 2.^50^, ^51^ Due to its ability to cross‐present antigens and trigger an antitumor immune response, cDC1 is often referred to as cross‐presenting DCs. It presents tumor antigens to CD8^+^ Tn cells via MHC class I (MHCI), which activates the initial immune response.^52^, ^53^ Upon activation, CD8^+^ Tn develops and differentiates into immune memory cells, such as stem cell‐like memory T cells (TSCMs), central memory T cells (TCMs), effector memory T cells (TEMs), and T‐terminal effectors (TTEs). This induces the occurrence of recall responses by the restimulation of past antigen encounters. The quick responses to the secondary stimulation of antigens form numerous specific immune cells immediately and effectively kill tumor cells.^54^, ^55^ It is worth noting that only DCs among several APCs can stimulate the initial immune response and induce Tn to produce an immune response targeting “new” antigens. It is speculated that the damage of DCs is closely related to refractory tumors. In addition, cDC1 plays a vital role in lung immunity,^56^ while cDC2 is a major subpopulation of DCs in different human tissues and organs. Besides, cDC2 presents tumor antigens to CD4^+^ Tn cells via the MHCII and activates the initial immune response of CD4^+^ Tn. Similar to CD8+ Tn cells, CD4+ Tn ones undergo activation and subsequently differentiation into various types of immune memory cells, including TSEMs, TCMs, TEMs, TTEs, and so forth. This process establishes a memory immune response that is of significance to orchestrate immune reactions and maintain homeostasis.^56^ Moreover, pDCs are key players in antiviral immunity. In pDCs, type I IFN is produced in large amounts, which accelerates innate and adaptive immunities^57^ (Figure 1). The IFN is regarded as the key natural immune defense of the body against pathogen infection. Furthermore, it regulates immunity and inhibits tumor cell proliferation.^32^, ^58^

### 2.3 DCs interact with T cells at the immune synapse

The primary mechanism by which DCs stimulate T cells is through their interaction, and the formation of immune synapses is a pivotal element of this process. Immune synapses denote a specialized structure that develops at the interface of cell–cell contact during the interaction between APCs and T cells.^59^, ^60^ Within this process, DCs provide three critical signals.

The first signal arises when T cells recognize analogous peptide antigens displayed on the surface of DCs through T cell receptors (TCRs).^61^, ^62^, ^63^, ^64^ MHCI molecules bind to CD8^+^ Tn cells, while MHCII molecules bind to CD4^+^ Tn cells to present specific antigens to Tn.^65^ The interaction involving MHCs, antigens, and TCRs triggers the activation of T cells, which initiates downstream signals through the immune receptor tyrosine‐based activation motif.^66^ The second signal can be classified into co‐stimulatory and co‐inhibitory molecules based on their distinct effects.^67^, ^68^ CD28, inducible co‐stimulator, tumor necrosis factor receptor superfamily member 4 (OX40), and CD40L are co‐stimulatory molecules. All these pivotal signaling molecules are accountable for the activation, differentiation, and survival of T cells. Co‐stimulatory signals collaborate with MHC–antigen–TCR complexes to augment the activation of T cells. Among them, CD28 is crucial in the immune response of T cells.^69^, ^70^ After the activation of CD28, phosphatidylinositol‐3‐hydroxykinase/Akt (PI3K/Akt) and other signals are induced to facilitate the proliferation and development of Tn. As a result, Tn–TSCM–TCM–TEM–TTE forms the initial immune response and memory immune response, which allows the body to combat both the “old” antigens produced by tumor recurrence and the “new” ones generated by tumor variation.^71^, ^72^ After CD28 activation, signaling pathways like PI3K/Akt^73^ and nuclear factor‐κB^74^ are activated, which contributes to completing the development, differentiation, and proliferation of Tn and Tm. This results in effector T cells that can eliminate tumors and other pathogens. On the contrary, CTLA‐4 competes with CD28 for the binding of CD80/86 on DCs, while PD‐1 binds to PD‐L1 on the surface of DCs, which inhibits the signals activating it.^75^, ^76^ Both positive and negative co‐stimulatory molecules are important to initiate, regulate, and terminate the immune response effectively.^77^ DCs, a third signal, secrete cytokines in response to signals from the MHC antigen TCR complex and sufficient co‐stimulation.^78^, ^79^ In collaboration with T cells, DCs can secrete abundant IL‐12 and IL‐18. Furthermore, they are the primary partners of T cells and indispensable for activating the proliferation of T cells, inducing the production of CTLs, orchestrating the priming of Th1 type immune response, and enhancing tumor elimination.^80^, ^81^

Of note, T cells are activated by DCs through the presentation of antigens and the provision of immunomodulatory signals via cell–cell contact and cytokines. DCs possess the capability of identifying, engulfing, and processing tumor antigens. Once processed and presented, antigens are presented to CD4^+^ or CD8^+^ T cells via MHCI or MHCII molecules displayed on the surface of DCs, which initiates T cell activation. Mature DCs exhibit CD80, CD86, CD40, and other high‐level co‐stimulatory molecules, which thereby supply a second signal for complete T cell activation. DCs also secrete a variety of cytokines such as IL‐2, IL‐12, and IFN‐γ, which further stimulate the proliferation and differentiation of activated T cells and thus motivate the initiation of the immune response. Unlike the activation of memory T cells, that of naive T cells relies on the presence of DC stimulation signals to a greater extent. Hence, DCs as the most potent mature APCs possess a unique ability to prime naive T cells.

## 3 FUNCTION OF DCS AND T CELLS IN NSCLC

### 3.1 T cells affect the prognosis and efficacy of immunotherapy in NSCLC patients

The number of lymphocytes in circulation, including both proportion and absolute count, is a crucial indicator of the effectiveness and stress levels of the immune system.^82^ Different cells within the immune system interact and mutually influence each other, with the appropriate ratio of immune cells being crucial for their functionality. Petersen et al. confirmed that higher levels of Treg cells increase the risk of recurrence, and detected Treg cells in half of NSCLC patients.^83^ In addition, Liu et al. also suggested that targeting Treg cells can be an interesting method for the immunotherapy of lung cancer.^84^

Clinically, the absolute number of lymphocytes is much more important than their proportion. Tumors can inhibit the proliferation of bone marrow cells during the developmental period, which significantly decreases the absolute number of various immune cells, including lymphocytes.^85^, ^86^ Meanwhile, one main consequence of chemotherapy and radiotherapy is the damage to the bone marrow,^87^ which thus decreases the absolute number of lymphocytes.^88^, ^89^ Low lymphocyte absolute count is an independent negative prognostic factor for advanced malignant tumors.^90^ Oh et al. found that the baseline level of absolute lymphocyte count is an independent prognostic factor of rectal adenocarcinoma patients who received preoperative radiotherapy and chemotherapy in different treatment and follow‐up periods.^91^ Furthermore, Xia et al. found that the absolute rather than relative number of lymphocyte subsets in NSCLC patients was closely associated with the period of disease progression and progression‐free survival. Lymphocyte subsets served as biomarkers for disease prognosis and efficacy.^92^

T cells can also be effective in reducing NSCLC by increasing their activity. The activated tumor antigen‐specific CD8^+^ T cells can recognize and secrete cytotoxic molecules to kill tumor cells.^93^ As claimed by Wu et al., the Fuzheng anticancer formula effectively enhances the secretion of IFN‐ γ by CD8^+^ T cells, which is critical in clearing tumor cells.^94^ As the most studied checkpoint pathways, CTLA‐4 and PD‐L/PD‐lL inhibit the activity of T cells in different ways.^95^, ^96^ The proliferation of T cells is regulated by CTLA‐4 early in the immune response,^97^ whereas the suppression of T cells is mediated by PD‐1 later in the immune response.^98^ The ICIs, including ipilimumab, tremelimumab, nivolumab, pembrolizumab, atezolizumab, and durvalumab, have opened a new era in the treatment of advanced NSCLC.^99^, ^100^

### 3.2 DCs play an important role in NSCLC

DCs, an essential antigen‐presenting cell, play a pivotal part in determining the prognosis of NSCLC.^101^ The progression‐free survival of NSCLC shows a positive correlation with the proportion of DCs. The infiltration of DCs in NSCLC is linked to the expression of specific genes like toll‐like receptor 3 (TLR3) and topoisomerase IIα, considering the gene expression characteristics and DC infiltration in NSCLC patients maks it possible to develop effective strategies for treating patients with refractory cancer.^102^ In NSCLC, pDCs and myeloid DCs (mDCs) are primary DCs.^103^ Zahran et al. and Wang et al. demonstrated that pDCs in the peripheral blood of NSCLC patients are related to good prognosis, lower tumor stage, and longer mean OS time, while mDCs are the opposite.^101^, ^104^ Bianchi et al. discovered that the activation of CD8^+^ T cells and the proportion of TLR3‐CD1–3+ DCs positively correlate with OS.^105^ NSCLC patients exhibit a significantly higher maturation rate of DCs compared with healthy individuals. Poor tumor prognosis has the following characteristics: the absence of functional DCs within lung tumor lesions, the recruitment of pDCs to surrounding lung tumor tissues, lung tumor‐induced regulatory DCs, the underexpression of DC effector molecules in lung tumors, and the secretion of immunosuppressive molecules by infiltrating DCs in lung cancer tissues.^106^

## 4 DEVELOPMENT TREND OF CLINICAL IMMUNOTHERAPY BASED ON DCS AND T CELLS

For advanced NSCLC, ICIs are an important treatment. A new modality for the treatment of blood cancer has emerged, namely chimeric antigen receptor T cells (CAR‐T). However, the low efficacy of ICIs in treating solid tumors limits their application. Despite having been reported, CAR‐T is still in the exploratory stage.^107^, ^108^ Advances in immunotherapy are combined with those in the understanding of immune cells, especially DCs, to find that the trend of next‐generation immunotherapy is moving towards developing artificial DCs and CAR‐T that can be used to treat solid tumors. Both can override the limitation of low immunity in tumor patients.

### 4.1 Engineered DC is the latest treatment strategy for NSCLC

Artificial APCs (aAPCs) are alternative options to custom‐made autologous APCs that effectively stimulate antigen‐specific T cells in vitro. These aAPCs can be easily prepared from premade components.^109^, ^110^, ^111^ Cell‐based aAPCs,^112^ which are generated through retroviral or lentiviral transduction, have been extensively studied in different cell lines such as fly black stomach cells, NIH3T3 (National Institutes of Health 3T3)^113^ mouse fibroblasts and K562 human erythroid leukemia cells.^114^ These cells can be easily obtained from source companies and distributors and stored for extended periods.^115^, ^116^ Due to their ability to regulate signal delivery, aAPCs emerge as an appealing choice. Their allure lies in the relatively uncomplicated preparation process, which includes microlatex, polyethylene glycol, magnetic beads, and lipid‐based vesicles.^117^ In general, the focus of the aAPC approach has been on MHCI stimulation to induce the generation of CD8^+^ CTLs, as these cells hold the capacity for the antigen‐specific lysis of tumor cells.^118^, ^119^ The development of aAPCs with diverse sizes, shapes, surface ligand distribution, and ligand mobility showcases the efforts to simulate different aspects of natural DCs.^116^ These structural variations among cell‐free aAPCs exert an impact on the extent of T cell activation.

It has been demonstrated that DCs often display inadequate maturity within the tumor microenvironment and reduced effectiveness in presenting tumor antigens.^120^ Consequently, one crucial approach to develop DC vaccines is to target the delivery of antigens and adjuvants to cells in vivo. To address this issue, Sun et al. developed intelligent artificial DCs (iDCs) that retain antigen‐presenting ability and T cell initiation function. The membrane of DCs on the surface of iDCs can effectively present antigens.^120^ Upon injection into mice, iDCs migrate to lymph nodes and stimulate the activation and proliferation of T cells. Cancer‐specific T cells undergo TCR‐dependent activation, which results in the targeted elimination of tumor cells upon the recognition of antigens presented by MHC receptors. Additionally, the activated T cells secrete cytokines like TNF‐α, which reduces the expression of heat‐shock proteins in tumor cells and thus enhances the sensitivity of tumor cells to heat stress. Subsequently, mild photothermal treatment is applied to eradicate the remaining tumor cells by subjecting them to temperatures ranging from 42°C to 45°C. It should be noted that this treatment also induces the death of immunogenic cells, which triggers the activation of DCs of the body and rejuvenates the tumor immune cycle. By combining the advantages of DC immunotherapy and photothermal therapy, iDCs serve as a novel and precise antitumor nanosystem significantly enhancing the antitumor immune response of the body, which thereby improves the effectiveness of tumor treatment. This approach provides a promising strategy for immunosensitized low‐temperature photothermal therapy.^121^ In addition, Suarez et al. developed the technology of artificial immune modulation nanoparticles (AIM NPs). Through a naturally occurring signaling mechanism, AIM NPs act as a synthetic APC and directly bind to target T cells. To transmit signal 1, the peptide‐loaded HLA class I dimerization fusion protein presents the antigen peptide to homologous TCRs. On the other hand, signal 2 is transmitted by monoclonal antibodies against CD28 receptors, which transmit co‐stimulating signals also known as “danger signals” to induce the activation and proliferation of antigen‐specific T cells.^122^

The antigen presentation of DCs without functional “arms” weakens the DC–T cell interaction, which disrupts T cell induction.^123^ A common strategy for activating T cells is to modify DCs to enhance their antigen presentation. To regulate the interactions between different cell types, synthetic ligands or receptors can be “engineered” or modified on cell surfaces.^124^, ^125^ By virtue of their specificity and bond stability, lectins play an important role in promoting intercellular recognition and adhesion.^126^ Recent research has shown that DCs modified with sugar polymers can specifically adhere to T cells via carbohydrate lectin binding, which enhances the stability of DC–T cell binding and facilitates T cell activation. Therefore, it is valuable and effective to design and optimize cell vaccines by adding appropriate synthetic sugar polymers to cell surfaces.^127^, ^128^

### 4.2 Another new strategy for treating NSCLC is CAR‐T therapy

As a kind of genetically engineered T cells, CAR‐T cells express synthetic CAR vectors. These CAR‐T cells specifically recognize and bind antigens like CD19 on tumor cells, and effectively kill tumor cells.^129^, ^130^ Furthermore, CAR‐T therapy has emerged as a novel treatment strategy with promising results against blood tumors.^131^ Nevertheless, CAR‐T therapy failed to get satisfactory results when research further turned its attention to solid tumors, which take up 90% of all tumors. Solid tumors themselves and their microenvironment are particular, which pose great challenges to CAR‐T therapy.^132^

Unlike CD19, tumor cells in solid tumors typically express multiple targets abnormally, and these abnormally expressed antigens are expressed in normal tissues as well. For example, the current research targets for glioma include prostate‐specific and carcinoembryonic antigens (CEAs), human epidermal growth factor receptor 2 (EGFR2), epithelial cell adhesion molecules, and mesothelin.^133^, ^134^ Among the most abundant and critical components of the tumor microenvironment are cancer‐associated fibroblasts,^135^ which constitute the tumor stromal layer releasing certain inhibitory cytokines. Immunosuppressive cells such as Treg cells, bone marrow‐derived suppressive cells, and M2‐type macrophages secrete cytokines like TGF‐β and IL‐10 to negatively regulate CAR‐T cell immune response. In addition, solid tumor cells lose cytokine receptors and escape immune cell surveillance. CAR‐T cells are not effective in responding to chemotaxis secreted by tumor cells, which inhibits their homing ability. However, the high expression of immunosuppressive receptors in solid tumors inhibits the effective activation of CAR‐T cells and lowers the efficacy of CAR‐T therapy.^136^, ^137^, ^138^

CAR‐T therapy, which has emerged as a hopeful strategy for treating NSCLC, achieves significant breakthroughs and enters a phase of rapid development.^139^, ^140^ Thus far, more CAR‐T studies have focused primarily on NSCLC.^141^, ^142^ The most common target antigens of NSCLC include human EGFRs, mesothelin, mucin 1, PD‐L1,^143^ CEAs,^144^ and HER2.^145^ As a result, these antigens are potentially novel targets for NSCLC treatment.^146^, ^147^

## 5 CONCLUSIONS AND PROSPECTS

Immunotherapy for NSCLC based on DCs and T cells is still in the development stage and has been clinically proven to be effective against certain cancers. Recent studies have shown that the interaction between DCs and T cells is an emerging strategy for treating NSCLC. Unraveling the significance of DC–T cell interaction in NSCLC serves a dual purpose: It sheds light on the underlying mechanism of this cancer and offers valuable insights for its treatment. Moreover, engineered DC and CAR‐T therapies hold great promise for treating solid tumors by leveraging this understanding of DC–T cell interplay.

## AUTHOR CONTRIBUTIONS

Shuangcui Wang designed the review, prepared the figures, and wrote the manuscript. Guan Zhang and Qian Cui participated in the study's conception and design. Yanjie Yang and Dong Wang conducted the literature search. Aqing Liu and Ying Xia were involved in the conception and design of the study and revised the manuscript. Wentao Li and Yunhe Liu provided valuable comments. Jianchun Yu revised the manuscript. All authors made equal contributions.

## CONFLICT OF INTEREST STATEMENT

The authors declare no conflict of interest.
